# Age-Dependent Analysis of Suicidal Ideation, Suicide Attempts, and Suicides Associated with SSRI and SNRI Drugs Based on Pharmacovigilance Data

**DOI:** 10.3390/ph17121714

**Published:** 2024-12-19

**Authors:** Daria Schetz, Jacek Sein Anand, Łukasz Sein Anand, Ivan Kocić

**Affiliations:** 1 Department of Pharmacology, Faculty of Medicine, Medical University of Gdańsk, 80-210 Gdańsk, Poland; ikocic@gumed.edu.pl; 2 Pomeranian Centre of Toxicology, 80-104 Gdańsk, Poland; jacek.anand@gmail.com (J.S.A.);; 3 Department of Clinical Toxicology, Medical University of Gdańsk, 80-210 Gdańsk, Poland

**Keywords:** antidepressants, SSRI, SNRI, psychiatric adverse drug reactions, suicidal ideation, suicidal attempts, suicides, pharmacovigilance

## Abstract

**Background:** Antidepressants such as SSRIs and SNRIs are widely prescribed; however, significant concerns exist regarding psychiatric adverse drug reactions (ADRs), particularly suicidal ideation, suicide attempts, and completed suicides. This study analyzes pharmacovigilance (PhV) data from the EudraVigilance database to assess the frequency of psychiatric ADRs, including suicide-related events, associated with six commonly used antidepressants. Another aim of the study is to evaluate the utility of pharmacovigilance data in providing insights into real-world risks associated with medications, highlighting the importance of improving the ADR reporting system and ensuring the completeness and reliability of ADR reports. **Methods:** Data from December 2001 to September 2024 were analyzed for duloxetine, citalopram, escitalopram, fluoxetine, venlafaxine, and sertraline. Reports were categorized by age, gender, and source, focusing on psychiatric ADRs and suicide-related events, including completed suicides and suicide attempts. **Results:** Psychiatric ADRs accounted for a substantial portion of total reported ADRs for the studied antidepressants, ranging from 33.9% to 38.2%. Venlafaxine had the highest count of psychiatric ADRs (13,134 cases), with duloxetine showing the highest relative percentage (38.2%). Completed suicides were most frequent with venlafaxine (1635 cases), while the highest percentage relative to total ADRs was observed for fluoxetine and citalopram (6%). ADRs occurred more frequently in women, particularly for duloxetine (67%) and sertraline (61.3%), and suicide attempts were prevalent in patients aged 18–64, with notable incidence in the 0–17 age group. **Conclusions:** This study highlights the significant patterns, risks, and underreporting of psychiatric ADRs associated with commonly prescribed antidepressants. Using EudraVigilance data and a worst-case scenario approach, it reveals the extent of suicide-related ADRs, age and gender disparities, and the impact of incomplete reporting on risk assessment.

## 1. Introduction

### 1.1. Background: Growing Concerns Regarding the Safety of Antidepressants (SSRIs and SNRIs)

Suicide is one of the leading causes of death, with a rate of 534.3 per 100,000 person-years [1]. Depression is common among those who die by suicide in later life, and research suggests that up to 75% of these suicides might be preventable with effective depression treatment [2]. Regardless of this, scientists increasingly highlight the significant rise in antidepressant prescriptions, which doubled in a decade, despite many patients having only mild to moderate depression, where antidepressants may be ineffective. They point out that only a small percentage of patients truly benefit from these drugs, with most of the observed effects being placebo responses. Despite this, non-drug alternatives are often overlooked, and the long-term use of antidepressants is common, even though evidence supporting their long-term safety is limited. Furthermore, patients often experience withdrawal symptoms when discontinuing the medication, raising concerns about dependence. It is emphasized that antidepressants are overprescribed, lack real efficacy for many patients, and contribute to a cycle of overdiagnosis and overtreatment in mental health [3,4,5]. The available analyses conducted in recent years show that the most commonly prescribed antidepressants were selective serotonin reuptake inhibitors (SSRI), with citalopram being the most frequent (47.04%), followed by escitalopram (11.81%) and fluoxetine (0.55%), and serotonin–norepinephrine reuptake inhibitors (SNRI) including duloxetine (0.71%) and venlafaxine (1.54%) [6]. It is obvious that these drugs, due to their specific mechanisms of action, cause a wide range of side effects of varying degrees of severity.

In recent years, several studies have explored the association between the use of antidepressant drugs and the risk of suicide attempts and suicidal ideation. The conclusions suggest that, especially in the first weeks of treatment, these medications may increase the number of suicidal behaviors and suicide attempts in some patients. Recent studies indicate that the use of antidepressant drugs among children and adolescents has significantly increased in recent years [7] despite the FDA issuing a “black box” warning in 2004 for SSRIs regarding their potential link to suicidal thoughts and behavior in this age group [8]. This warning was later extended to include young adults aged 18–24 in 2007 [9]. However, it is also a fact that the undertreatment of depression is prevalent among children and adolescents, but evidence of its impact on suicide risk remains scarce.

During depression treatment, some patients experience new or worsening suicidal thoughts, termed TESI (treatment-emergent suicidal ideation) or TWOSI (worsening suicidal ideation). The occurrence of TESI varies across studies, ranging from 3.2% to 17%, depending on the population examined and the criteria used to define TESI [10]. Various genetic, sociodemographic, and clinical factors contribute to TESI and TWOSI, including early-onset depression, gender, the severity of the depression, physical pain, and poor response to antidepressants [11,12,13]. Despite these associations, it remains debated whether antidepressants directly increase suicidal thoughts and behaviors in children and adolescents. This debate is particularly relevant given the rising prevalence of antidepressant use in this group over the past decade [14].

A meta-analysis of randomized controlled trials (RCTs) has shown inconsistent results regarding antidepressants and suicidal risk. Earlier research found an increased risk with SSRIs in children [15]; however, more recent studies highlight venlafaxine as the only antidepressant associated with suicidal behavior or ideation in youth [16,17]. Most RCTs exclude participants with suicidal thoughts, limiting their findings on this issue [18]. In 2022, a team of researchers conducted an updated meta-analysis of observational studies to investigate the association between antidepressant exposure and the risk of suicide and suicide attempts in children and adolescents [19]. The meta-analysis suggests that antidepressant use may raise the risk of suicide in children and young adults. They concluded that since untreated depression is a major suicide risk, clinicians should closely monitor at-risk patients, especially for TESI and TWOSI, and consider non-drug treatments as alternatives. In addition, they noted that while s-ketamine and ketamine may reduce suicidal thoughts temporarily, their long-term safety in younger populations is still uncertain, highlighting the need for further exploration of treatment strategies for this group [19]. In another study, scientists developed a microsimulation model using evidence from multiple sources to evaluate how varying durations of antidepressant treatment influence suicide risk in a nationally representative sample of children and adolescents with major depressive disorder (MDD). The results showed that both suicide rates and suicide attempt risks declined with longer treatment durations compared to no treatment at all. The study suggests that 12–36 weeks of antidepressant treatment can substantially lower suicide and suicide attempt risks in children and adolescents with MDD [20].

Apart from that, older adults, particularly men, have some of the highest suicide rates worldwide [21,22]. While there is evidence that antidepressant therapy can reduce suicide risk in older populations, few studies focus on the oldest age groups. Clinical trials often exclude these individuals due to frailty, despite their unique treatment responses and higher levels of comorbidities [23]. In one study, researchers rightly noted that while treating depression is key to preventing suicide in older adults, there are limited studies on how different patterns of antidepressant use affect suicide risk in this group. Moreover, there are no large population-based studies on the effects of using antidepressants alongside other psychoactive drugs like sedatives or hypnotics, which are commonly prescribed and may increase suicide risk. They conducted a study aimed at exploring the relationship between patterns of antidepressant use and the risk of suicide and suicide attempts in older adults (aged 75 and above) in Sweden. The study showed that switching antidepressants and the use of anxiolytics or hypnotics may signal a higher risk of suicidal behaviors in older adults starting antidepressant therapy [24].

Another Swedish study investigated the link between antidepressant use and suicide rates in people aged 75 and older. Following over 1.4 million individuals from 2007 to 2014, the study found that one-third of those who died by suicide had been prescribed antidepressants in the last three months of life. Suicide rates were higher among antidepressant users, especially those taking mirtazapine compared to SSRIs. Antidepressant use contributed to about a quarter of the increase in suicide rates, highlighting the need for better prevention strategies in older adults at risk of suicide [25].

While various study findings highlight how many factors can contribute to the occurrence of psychiatric side effects of antidepressant drugs, including suicide attempts, clinical trials conducted on these medications have numerous limitations and fail to account for the diverse real-world variables.

### 1.2. Mechanism of Action: How Do the Pharmacological Effects of SSRIs and SNRIs Relate to Their Safety Profile?

SSRIs work by selectively inhibiting the reuptake of serotonin (a neurotransmitter), into the presynaptic neuron. This inhibition increases the concentration of serotonin in the synaptic cleft, which enhances neurotransmission and boosts mood regulation. The primary target of SSRIs is the serotonin transporter (SERT), and by blocking this transporter, SSRIs prevent the reabsorption of serotonin into the neuron that released it. This leads to an increased level of serotonin available to bind to the postsynaptic receptors. Over time, this increased serotonin availability is believed to improve depressive symptoms and regulate mood [26,27].

SNRIs similarly inhibit the reuptake of serotonin but also block the reuptake of norepinephrine, another neurotransmitter involved in mood regulation and arousal. By inhibiting the reuptake of both serotonin and norepinephrine, SNRIs increase the levels of both neurotransmitters in the synaptic cleft, which helps regulate mood and alleviate symptoms of depression. Like SSRIs, SNRIs act on specific transporters, the serotonin transporter (SERT) and norepinephrine transporter (NET), to inhibit the reabsorption of these neurotransmitters, thus enhancing their availability to receptors on the postsynaptic neuron [26].

The therapeutic action of SSRIs and SNRIs, based on increasing serotonin and/or norepinephrine levels, can lead to various side effects due to the complex role these neurotransmitters play in multiple physiological processes. For instance, excessive serotonin in non-targeted pathways can lead to side effects like gastrointestinal disturbances, sleep disturbances, or sexual dysfunction. Similarly, the norepinephrine activity in SNRIs may contribute to side effects such as increased heart rate or blood pressure. Moreover, the effects on neurotransmission can affect mood regulation in unpredictable ways, sometimes leading to an increased risk of suicidal ideation, particularly in younger patients during the initial weeks of treatment [28]. Suicide attempts at the beginning of treatment with SSRIs and SNRIs can be explained by the fact that these drugs initially improve the patient’s psychomotor activity, activating individuals who may have previously experienced psychomotor retardation due to depression [29]. Unfortunately, while physical activation occurs early, the improvement in mood and psychological well-being takes longer to manifest (from 4 to 6 weeks). This mismatch in timing can make it easier for individuals to act on suicidal thoughts as their ability to make decisions improves, even before their emotional state has significantly recovered [29,30].

Both SSRI and SNRI groups have several representatives. Although they share the characteristic mechanism of action typical for their respective groups (primarily inhibiting the reuptake of serotonin or both serotonin and norepinephrine), they differ significantly in their pharmacokinetics and in their additional effects, such as residual affinities for other transporters or receptors, such as, e.g., histamine receptors and muscarinic receptors. The pharmacokinetics of SSRIs and SNRIs, particularly their metabolism via liver enzymes, play a significant role in individual variability in drug response. Many SSRIs and SNRIs are metabolized by cytochrome P450 (CYP450) enzymes, with isoforms such as CYP2D6 and CYP3A4 being the most commonly involved. Polymorphisms in CYP2D6 can lead to differences in drug metabolism, classifying individuals as poor, intermediate, extensive, or ultra-rapid metabolizers. For example, poor metabolizers of CYP2D6 substrates may experience higher plasma concentrations of certain SSRIs (e.g., fluoxetine, paroxetine), increasing the likelihood of adverse reactions, including psychiatric ADRs. Potential drug–drug interactions also contribute to variability in psychiatric ADRs. For instance, the concurrent use of other CYP450 inhibitors, such as antifungal agents or macrolide antibiotics, can increase SSRI/SNRI plasma levels, heightening the risk of ADRs. Conversely, enzyme inducers, such as rifampin or carbamazepine, may reduce drug levels, potentially diminishing therapeutic efficacy and leading to treatment failure. These variations can influence the clinical response to treatment, as well as the profile of adverse effects. In summary, differences in metabolism, half-life, and the degree of interaction with other neurotransmitter systems mean that patients may experience varied therapeutic outcomes and side-effect profiles, even within the same drug class. Fluoxetine (Prozac), as the first and one of the most well-known SSRIs, has been a source of significant controversy, particularly due to its long half-life (t½ > 60 h) and its association with increased suicidal risks, especially in younger patients. During its clinical trials (phase III), there were reports of suicidal behavior, which raised concerns about its safety profile even before it was fully approved. Some studies indicated that fluoxetine, compared to other antidepressants, might lead to an increased risk of suicidality in certain patient populations [31]. Thus, selecting a specific agent must consider these pharmacological nuances to optimize treatment efficacy and minimize potential risks [32,33]. These pharmacokinetic considerations underline the importance of personalized medicine approaches in antidepressant therapy, including genetic testing for CYP450 polymorphisms and careful review of concomitant medications to optimize treatment outcomes and minimize ADR risk.

Key findings from previous studies on antidepressants and suicide risk:

- Suicide rates: Suicide is a leading cause of death, with a rate of 534.3 per 100,000 person-years [1].
- Impact of effective depression treatment: Up to 75% of suicides could be prevented with proper treatment for depression [2].
- Overprescription of antidepressants: Antidepressant prescriptions have increased significantly; however, they often have limited effectiveness in patients with mild to moderate depression [3,4,5].
- Most prescribed antidepressants: The most commonly prescribed drugs are citalopram (47.04%), escitalopram (11.81%), fluoxetine (0.55%), venlafaxine (1.54%), and duloxetine (0.71%) [6].
- Suicidal behaviors during initial treatment weeks: Antidepressants may increase suicidal behaviors during the first weeks of treatment, especially in children and adolescents. The FDA issued warnings about TESI and TWOSI risks in younger patients [7,8,9].
- TESI/TWOSI (suicidal thoughts): New or worsening suicidal thoughts occur in 3.2% to 17% of cases. Risk factors include early-onset depression, gender, severe depression, physical pain, and poor response to treatment [10,11,12,13].
- Controversy over SSRIs and SNRIs in youth: Studies show increased suicide risk in young patients, particularly with venlafaxine [15,16,17].

Antidepressants in older adults: Older men have some of the highest suicide rates. Mirtazapine is associated with higher suicide risk in this group [21,22,23,24,25].

Long-term antidepressant use: Long-term use raises concerns due to limited safety evidence and withdrawal symptoms [3,4,5].

Monitoring young patients: Close monitoring for TESI and TWOSI is recommended in children and adolescents, especially during the first weeks of therapy [19].

Mechanism increasing suicide risk early in treatment: Antidepressants can activate physical energy before improving mood, increasing the risk of acting on suicidal thoughts [29,30].

### 1.3. The Role of Regulatory Frameworks in ADR Reporting and Their Impact on Patient Safety

Robust regulatory frameworks play a pivotal role in monitoring and reporting ADRs. These frameworks provide clear guidelines for healthcare professionals and patients, facilitating the prompt identification of safety risks. The early detection of potential issues is essential for minimizing the risks associated with ADRs, enabling timely interventions such as safety updates, risk mitigation strategies, or product recalls to safeguard patient health. Efficient PhV systems are critical in achieving these objectives. Enhancements such as standardized reporting protocols and improved accessibility can substantially elevate the quality of PhV data, ultimately contributing to better patient safety outcomes. This study seeks to offer insights that could guide future regulatory improvements to address existing gaps and challenges in ADR reporting.

### 1.4. Aim and Scope of the Study

Given the rising number of suicides across various age groups in recent years, alongside concerns about untreated depression, the inefficacy of antidepressant treatments, and the emergence of psychiatric adverse effects following the initiation of antidepressants, we decided to analyze what conclusions can be drawn from PhV data. Unlike clinical trials, which often yield conflicting results, PhV data provide real-world evidence, offering valuable insights into the effects of these medications in broader, everyday use.

The aim of this study was to analyze data from the Eudravigilance database to assess the frequency and nature of reported adverse events associated with the most commonly used SSRIs and SNRIs, such as duloxetine, citalopram, escitalopram, fluoxetine, venlafaxine, and sertraline. Specifically, we examined the total number of ADR reports, the proportion of psychiatric adverse events, and the incidence of suicide attempts, suicidal ideations, completed suicides, and suicidal behaviors. We also sought to identify the age groups in which suicide attempts were most frequently reported and determine whether any of these antidepressants carried a higher suicide risk within specific age groups.

Furthermore, this study aimed to assess whether PhV data can provide sufficient information to draw conclusions about the relative risk of suicide associated with these drugs and whether they are useful for predicting drug safety risks, as well as identifying the limitations of such data.

### 1.5. The Importance of the Study for Clinical Practice and Public Health

This research offers a fresh perspective by analyzing real-world PhV data to explore the relationship between antidepressant use and suicidal ideation, attempts, and suicides, with a focus on age-specific trends. Unlike previous meta-analyses or clinical trials, which often exclude individuals with suicidal thoughts or rely on controlled populations, this study examines a broader and more diverse patient population. This approach provides a deeper understanding of the risks linked to SSRIs and SNRIs, especially in real-world settings where factors like comorbidities, concomitant medications, and age-related vulnerabilities significantly influence outcomes. The findings are highly relevant for both clinical practice and public health. For clinicians prescribing antidepressants such as SSRIs and SNRIs, understanding real-world data on adverse drug reactions (ADRs) is essential for making informed decisions. By analyzing PhV data from Eudravigilance, healthcare professionals can better anticipate the risks of these medications, particularly psychiatric adverse events. These data allow clinicians to balance the benefits of antidepressant treatment against potential risks, enabling them to provide safer, more effective care to patients, especially those at higher risk of suicide. Additionally, the study breaks new ground by addressing the limitations of PhV data, highlighting areas for improvement to enhance its reliability and usefulness in future research.

## 2. Results

### 2.1. Total Number of ADRs Reported for Antidepressive Agents

For all drugs studied, the vast majority of reports describing ADRs were provided by healthcare professionals (HP). The number of reports in which it was unknown who completed the report was not substantial, ranging from 10 to 142 reports (NS—non-classified) (Table 1).

For the analyzed medications, sertraline was associated with the highest number of ADRs (37,620 reported cases). Subsequently, the drugs most frequently reported to cause ADRs were, in order, venlafaxine (36,808 cases), duloxetine (33,438 cases), and escitalopram (25,058 cases). The least frequently reported adverse effects were associated with citalopram (22,413 cases) and fluoxetine (21,627 cases) (Table 2).

**Figure 1:**
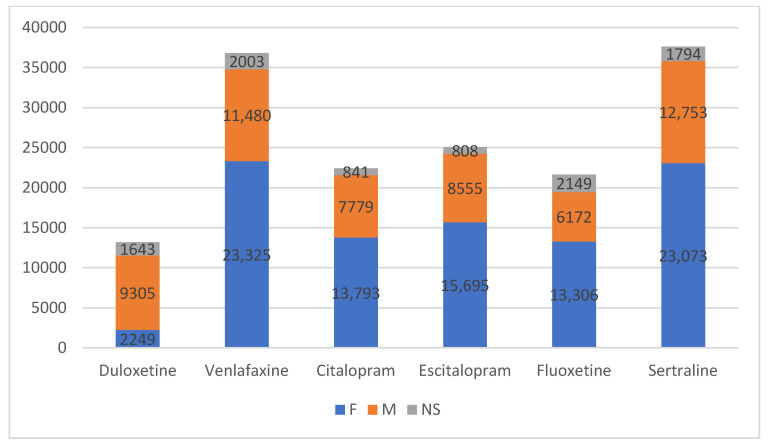
Distribution of ADRs by gender. Abbreviations and explanations used: F—females; M—males; NS—not specified.

For each of the studied medications, ADRs were most commonly reported in women. Specifically, duloxetine had the highest proportion of female reports (67%), followed by venlafaxine and escitalopram (both at 63%) and citalopram, fluoxetine, and sertraline, each with 61% of reports coming from women. Across all the drugs, adverse reactions were consistently reported more frequently in women than in men, with the percentage difference ranging from 29.5% to 39.5%. The percentage of reports with unknown gender ranged from 3.2% to 9.9%. Fluoxetine stands out with the largest proportion of unknown gender reports (9.9%), indicating a possible data gap that could affect gender-specific analysis for this drug (Figure 1).

### 2.2. The Frequency of Reporting Psychiatric ADRs to Antidepressant Drugs

Venlafaxine reported the highest number of psychiatric ADRs (13,134 cases). This was followed by duloxetine (12,790 cases) and sertraline (12,738 cases). Fewer reports described psychiatric ADRs for escitalopram (8907 reports), citalopram (8160 reports), and fluoxetine (8024 reports).

The percentage of psychiatric ADRs relative to total ADRs was highest for duloxetine at 38.2%, followed by fluoxetine at 37.1% and citalopram at 36.4%. Across all drugs, psychiatric ADRs consistently represented a significant portion of total ADRs, with values ranging between 33.9% and 38.2%. This suggests a substantial occurrence of psychiatric symptoms associated with these antidepressants (Table 2).

### 2.3. Reports Describing Psychiatric ADRs of Antidepressant Medications, in the Form of Deaths Due to Suicide, “Completed Suicide”

Venlafaxine, citalopram, and fluoxetine had the highest number of completed suicide reports with, respectively, 1635 cases, 1359 cases, and 1296 cases, while escitalopram had the fewest, with 880 cases. For duloxetine and sertraline, this adverse event was described in 1070 and 1049 reports, respectively.

The analysis of what percentage of all reported ADRs for a given drug were “completed suicides” revealed that for:

- Duloxetine, completed suicides accounted for 3% of all ADRs;
- Venlafaxine, completed suicides accounted for 4% of all ADRs;
- Citalopram, completed suicides accounted for 6% of all ADRs;
- Escitalopram, completed suicides accounted for 3.5% of all ADRs;
- Fluoxetine, completed suicides accounted for 6% of all ADRs;
- Sertraline, completed suicides accounted for 3% of all ADRs.

### 2.4. Reports Describing Psychiatric ADRs of Antidepressant Medications, in the Form of Suicidal Behavior

In the case of duloxetine, there were the most reports of suicidal behaviors, with 101 cases, 3 of which resulted in the death of the patient. The situation was very similar for sertraline, where there were 97 reports of suicidal behavior following the drug, including 3 deaths. Next in line were venlafaxine, fluoxetine, and escitalopram, which were mentioned, respectively, in 87 (3 were fatal), 83 (1 was fatal), and 66 (4 was fatal) reports as drugs that caused suicidal behavior. Suicidal behavior was least frequently mentioned for citalopram (36 cases, 0 fatal). Although death was rarely described in reports as a consequence of suicidal behavior, this was often because there was no information in the report to that effect—the effect of suicidal behavior was not known because it was not recorded by the person reporting the ADRs. In 13 to 60 reports, the final outcome of the adverse event of suicidal behavior was not known and therefore it was not possible to determine whether the suicidal behavior resulted in the patient’s death (incomplete completion of the reports) (Table 3).

### 2.5. Reports Describing Psychiatric ADRs of Antidepressant Medications, in the Form of Suicidal Ideation

Suicidal ideation was most frequently reported for duloxetine (3826 reports, including 6 fatal outcomes), followed by venlafaxine (1594 reports, including 31 fatal outcomes) and sertraline (1357 reports, including 17 fatal outcomes). This was followed by escitalopram (1148 reports, including 28 fatal outcomes) and fluoxetine (1011 reports, including 12 fatal outcomes). The least common suicidal ideation was reported for citalopram (731 reports, including 18 fatal outcomes). In as many as 259 (citalopram) to 2891 (duloxetine) reports, there was no information about the effect of the adverse reaction, i.e., whether it ended in the patient’s death or recovery (incomplete completion of the reports) (Table 3).

### 2.6. Reports Describing Psychiatric ADRs of Antidepressant Medications, in the Form of Suicide Attempts

The highest number of suicide attempts was recorded for venlafaxine (1583 reports), of which 52 cases were fatal, and in as many as 818 cases, the report lacks information on whether the adverse event was fatal or whether the patient survived.

The second drug with the highest number of suicide attempts was duloxetine (1359 reports), including death as an end result in 11 patients. Unfortunately, in as many as 901 reports, there was no information about whether the adverse event was fatal or whether the patient survived. Drugs that were slightly less frequently mentioned as causing suicide attempts were escitalopram (1190 reports, including 11 fatalities, and in 675 there was no information on the final outcome), sertraline (1123 reports, including 16 fatalities, and in 596 there was no information on the final outcome), and fluoxetine (1005 reports, including 51 fatalities, and in 507 there was no information on the final outcome). Citalopram was the least frequently mentioned drug as causing suicide attempts (930 reports, including 17 fatalities, and in 536 there was no information on the final outcome) (Table 3).

#### 2.6.1. Patient Age and Frequency of Suicide Attempts

For each drug studied, suicidal attempts were most frequently reported in the age group 18–64 years and least frequently in the 85+ age group.

The frequency of suicide attempts in the 0–17 age group varied depending on the drug:

- Suicide attempts were most frequently reported for sertraline (245 cases, accounting for 21.8% of all reports for this drug).
- Fluoxetine ranked second with 194 reports (19.3%), highlighting its significant presence in the younger population.
- Escitalopram came third, with 175 cases (14.7%).
- The fewest cases in this age group were reported for duloxetine (29 reports, accounting for only 2.1% of all reports for this drug).
- Overall trends: For most drugs, the percentage of suicide attempts in the 0–17 age group represents a notable proportion of all reports, indicating the need for particular attention when these drugs are prescribed to children and adolescents.

#### 2.6.2. Prevalence of Missing Age Information in Reports of Suicide Attempts Across Antidepressants

Reports describing suicide attempts frequently lacked information about the patient’s age, with the highest proportion observed for duloxetine, where the age of the patient was missing in 34% of cases. For the remaining drugs, the number of reports in which the age of the patients was unknown ranged from 16 to 25% (Table 4).

### 2.7. Reports Describing Suspected Suicide as a Consequence of Antidepressants

The drug that was mentioned in the greatest number of reports as suspected of causing fatal suicide was sertraline (74 cases). The second such drug was escitalopram (67 cases) and the third was fluoxetine (63 cases). Duloxetine (60 cases) and venlafaxine (56 cases) were mentioned slightly less frequently. Citalopram was least frequently mentioned as a drug suspected of causing suicide—50 cases. It is worth emphasizing that all reports were submitted exclusively by healthcare professionals (Table 2).

### 2.8. The Number of Suicide-Related Reports in the Form of Suicidal Behaviors, Suicidal Ideations, and Suicide Attempts, Where the Outcome of the ADRs Remains Unknown

In our analysis of the psychiatric ADRs associated with the six antidepressants, a significant number of reports lacked crucial outcome information for suicidal behaviors, suicidal ideations, and suicide attempts. This absence of data, due to incomplete reports, made it impossible to determine whether these events resulted in death, hospitalization, or recovery.

For duloxetine, there were 3852 reports where the outcome of suicide-related ADRs remained unknown. This drug had a relatively high proportion of reports lacking outcome data compared to the others, suggesting a considerable number of potentially serious yet unresolved cases.

Venlafaxine showed 1627 reports where the outcome of ADRs was unknown. Despite having the highest number of completed suicide cases (1635), this drug also exhibited a large number of incomplete reports, limiting the ability to fully assess the severity of these psychiatric ADRs.

Citalopram had 808 cases of unknown outcomes for suicidal behaviors, ideations, and attempts. While the total number of psychiatric ADRs was lower compared to venlafaxine and duloxetine, this still represents a substantial gap in outcome data.

For escitalopram, 1128 reports of suicide-related ADRs were incomplete. This number is notably higher than that of citalopram, despite fewer overall psychiatric ADRs being reported for escitalopram.

Fluoxetine had 1033 reports where the outcome was unknown, showing a similar trend of incomplete data. Despite being one of the drugs with fewer psychiatric ADRs overall, the incomplete outcome data raises concerns about the potential underestimation of serious ADRs.

Lastly, sertraline had 1224 reports where the outcomes of the above-mentioned ADRs remained unreported. Although sertraline had the highest total number of psychiatric ADRs (12,738), it also exhibited a relatively high number of cases with unknown outcomes, further complicating the risk assessment for this drug (Table 3).

These findings suggest that incomplete reporting of the outcomes in a significant number of cases across all drugs makes it difficult to draw definitive conclusions about the full scope and severity of suicidal behaviors and related ADRs. The missing data limit our ability to accurately assess the potential fatality or recovery rates associated with these antidepressants.

### 2.9. Estimating the Risk of Suicidal Behavior, Suicidal Ideation, Suicide Attempt, and Suicide Based on Past PhV

The drug most frequently mentioned for causing psychiatric ADRs, completed suicides, and suicide attempts was venlafaxine.

Duloxetine was the second most frequent drug to cause psychiatric ADRs, and additionally, it was the leading drug among the others in causing suicidal behavior and suicidal ideation. Notably, this drug was also the second most frequent (after venlafaxine) in causing suicide attempts. Interestingly, completed suicides were reported the least commonly for this drug compared to the other medications.

The drug that most often caused ADRs of all categories, not only psychiatric, was sertraline. In fact, it was the third most common drug causing psychiatric adverse events, (they accounted for 34% of all reported ADRs of this drug). However, completed suicide was noted less often than after venlafaxine, citalopram, fluoxetine, and duloxetine.

Citalopram was listed as the second most frequent drug (after venlafaxine) to cause completed suicides. However, suicide behaviors, suicidal ideations, suicide attempts, and suspected suicides occurred the least frequently compared to the other drugs.

Completed suicides occurred the least frequently with escitalopram. Suicidal ideations and suicidal behaviors were relatively rare compared to the other drugs. Unfortunately, this drug was the second antidepressant in the analysis suspected of causing suspected suicides.

The fewest reports describing all ADRs and all psychiatric ADRs were recorded for fluoxetine. This drug was never listed as the first or second most frequent drug causing completed suicides, suicidal behaviors, suicidal ideations, or suicide attempts. Suspected suicides were reported with this drug as moderately common. However, completed suicides accounted for 6% of all reported ADRs for this medication, which, along with citalopram, is the highest among the other drugs.

### 2.10. Incidence of Serious Suicidal ADRs with SNRIs and SSRIs: A Comparative Analysis Dased on a Six-Level Color-Scale Classification of ADR Frequency

Comparing the number of reports describing completed suicides, suicidal behaviors, suicidal ideations, suicide attempts, and suspected suicides across the drugs included in the analysis, it can be observed that brown and red labels (indicating the two most frequently occurring ADRs among the other drugs) appear most often with the SNRIs—venlafaxine and duloxetine. Among the SSRIs, sertraline stands out, as the alarming brown or red labels were used twice. Neither brown nor red labels were used for fluoxetine, and for citalopram and escitalopram, the red label appears only once.

### 2.11. Worst-Case Scenario Estimation

The estimated total number of ADRs, psychiatric ADRs, and completed suicides (fatal cases) varied across the analyzed antidepressants in the worst-case scenario, assuming only 6% of ADRs were reported.

Sertraline had the highest estimated total number of ADRs, reaching 627,000, as well as 212,300 psychiatric ADRs, but it ranked lower for completed suicides, with 17,483 cases. Venlafaxine followed, with 613,467 total ADRs, 218,900 psychiatric ADRs, and the highest number of completed suicides at 27,250 cases.

Duloxetine had the third highest number of total ADRs (557,300) and psychiatric ADRs (213,167) but fewer completed suicides (17,833) compared to venlafaxine and citalopram. Citalopram, in contrast, had 373,550 total ADRs and 136,000 psychiatric ADRs; however, it ranked second in completed suicides, with 22,650 cases.

Fluoxetine and escitalopram had comparatively lower estimated numbers. Fluoxetine accounted for 360,450 total ADRs, 133,733 psychiatric ADRs, and 21,600 completed suicides, while escitalopram had 417,633 total ADRs, 148,450 psychiatric ADRs, and the lowest number of completed suicides at 14,667 cases.

These results indicate that venlafaxine was associated with the highest number of completed suicides, while escitalopram had the lowest. For total ADRs, sertraline ranked the highest and fluoxetine the lowest among the analyzed antidepressants. This variability underscores the differing safety profiles and potential risks associated with these medications (Table 5).

## 3. Discussion

Our study revealed that, among all patients treated with the six most popular antidepressants, completed suicides were reported in between 880 and 1635 patients, depending on the specific drug. On one hand, considering the large number of patients using antidepressants in Europe, this number of suicides may appear proportionally modest. However, it is crucial to remember that, as numerous studies have shown, the reporting of ADRs is significantly underreported worldwide, including in Europe. For instance, it is estimated that only 6–10% of all ADRs are reported. A European systematic review found that the median rate of underreporting of ADRs by medical staff was 94 % [34,35,36]. Given these data, it is important to acknowledge that the actual number of psychiatric ADRs associated with antidepressants is significantly higher. We attempted to estimate this number by applying the worst-case scenario analysis, assuming the least optimistic yet highly plausible scenario that only 6% of ADRs are reported. Based on this assumption, we estimated that the drug most frequently reported as causing completed suicides—venlafaxine—could have been responsible for as many as 27,250 completed suicide cases. Similarly, we estimated that the actual number of psychiatric ADRs for the antidepressants analyzed in our study could reach as many as 1,062,550 reports. This demonstrates that it is possible to approximate the true number of ADRs based on data reported in the EudraVigilance database.

It is logical that the greater the number of patients taking a given drug, the more the adverse effects caused by it should be observed. However, it should be remembered that reporting adverse effects is also subject to certain trends. Reporting new or severe, life-threatening adverse effects is strongly recommended, more so than reporting well-known and documented ones [37]. Therefore, there should be fewer reports describing the former type of ADRs. However, many studies show that healthcare professionals may be reluctant to report these most significant ADRs, which results in them not being reported more frequently. Research conducted in Poland suggests that the reluctance to report serious and fatal ADRs may stem from the fact that some healthcare professionals are unaware of the importance of such reports and are insufficiently prepared [38]. Furthermore, a 2015 study conducted in the Pomeranian Voivodeship of Poland found that physicians often fear being accused of improper prescription practices when they report the adverse effects of medications. This may explain why they feel more comfortable and are more likely to report mild or moderate adverse reactions, or simply avoid reporting altogether, as there are no significant consequences for failing to do so [39]. On the other hand, the obligation of pharmaceutical companies in Australia (where ADR reports are collected by the Therapeutic Goods Administration, which is the medicines and therapeutic regulatory body of the Australian Government Department of Health) to prioritize serious ADRs could hypothetically lead to the overreporting of serious reactions compared to mild or moderate ones.

The ADR profiles of SSRIs and SNRIs differ due to their distinct pharmacological mechanisms of action [40]. SSRIs are more frequently associated with adverse effects such as sexual dysfunction, which can significantly impact patient adherence to treatment. Sexual dysfunction, reported in up to 50% of SSRI users in some studies, often includes decreased libido, delayed orgasm, or anorgasmia. In contrast, SNRIs, due to their norepinephrine reuptake inhibition, are more likely to cause physiological side effects such as hypertension and increased heart rate. Hypertension has been particularly noted with venlafaxine, especially at higher doses, necessitating regular blood pressure monitoring during treatment [41]. For clinicians, these differences highlight the importance of tailoring antidepressant selection to the individual patient’s medical history and risk factors. For example, SSRIs may be less suitable for patients for whom sexual function is a priority, while SNRIs may pose challenges for individuals with pre-existing cardiovascular conditions. Understanding these distinct ADR profiles enables more informed prescribing decisions, enhancing both the safety and efficacy of antidepressant therapy. The discontinuation of SSRIs and SNRIs is often associated with withdrawal symptoms, collectively referred to as antidepressant discontinuation syndrome (ADS). These symptoms can include dizziness, nausea, insomnia, irritability, and, in severe cases, heightened anxiety or depressive symptoms, which may exacerbate suicidal ideation or behaviors in vulnerable individuals. The risk and severity of withdrawal symptoms vary by drug and depend on factors such as the medication’s half-life and the duration of treatment. For example, paroxetine and venlafaxine, due to their shorter half-lives, are more commonly associated with withdrawal symptoms compared to fluoxetine, which has a longer half-life [42]. Withdrawal symptoms may mimic a relapse of depression, leading to challenges in distinguishing between the effects of medication discontinuation and the re-emergence of the underlying condition. This underscores the importance of gradual tapering strategies during discontinuation to minimize risks. For clinicians, these findings highlight the need for careful planning when stopping antidepressants and the importance of monitoring patients for withdrawal symptoms and potential suicidal behaviors during and after discontinuation.

Taking the above into account, in order to better understand the scale of suicides associated with the use of antidepressants, it is most informative to compare their number with the total reported ADRs of a given drug. Our study demonstrated that psychiatric adverse reactions accounted for as much as 36% to nearly 40% of all reported adverse reactions, underscoring the significance and challenges posed by these issues. Importantly, our study clearly demonstrated that even when the total number of reported ADRs was the highest in the case of a drug, the total number of psychiatric ADRs and completed suicides was not the highest (as was the case with sertraline).

The limitation we encountered while analyzing the collected reports was that they did not include information about the mental state of the patients before starting antidepressants, whether they had suicidal thoughts or attempted suicide before starting treatment. Certainly, some of the reports concern such patients, and supplementing the reports with such data would allow for drawing additional conclusions. According to a study conducted by Lopez-Castroman et al. [43], who investigated the short-term response to a new antidepressant treatment of 4041 depressed outpatients depending on their suicidal status, suicidal patients were less likely to improve or attain remission after drugs but not more likely to worsen than nonsuicidal patients. If the results of this study are reflected in the general population, then we must assume that some of the psychiatric adverse events we described were not so much caused by the drug but by the lack of therapeutic effect of the drug. It should be emphasized here that currently, the term adverse drug reaction also includes the lack of therapeutic effect of the drug. This broader definition is crucial for interpreting reports of antidepressant efficacy and safety, as it acknowledges that in some cases, what is perceived as an ADR may actually reflect the medication’s inefficacy, particularly in patients who were already suicidal before treatment. This could have a significant impact on evaluating antidepressant safety. In another study conducted by Jakobsen et al. [44], the relationship between the use of any antidepressants or only SSRIs and the risk of repeated suicide attempts was analyzed. The aim of the study was to determine whether antidepressant treatment was associated with an increased risk of recurrent suicide attempts. After analyzing data from 1842 individuals who had attempted suicide, the authors found that, after accounting for the baseline risk of repetition, the use of antidepressants was not significantly associated with an increased risk of repeated suicide attempts.

### 3.1. The Frequency of Psychiatric ADRs of the Drugs Studied

Our study demonstrated that ADRs are frequently reported for the antidepressants selected for this analysis. Moreover, a significant portion of these ADRs were psychiatric in nature. The percentage of psychiatric ADRs relative to total ADRs was highest for duloxetine at 38.2%, followed by fluoxetine at 37.1%, and citalopram at 36.4%. Across all the drugs, psychiatric ADRs consistently made up a substantial portion of total ADRs, ranging from 33.9% to 38.2%. This highlights a considerable occurrence of psychiatric symptoms associated with these antidepressants.

It is worth highlighting that citalopram was listed as the second most frequent drug (after venlafaxine) to cause completed suicides. However, suicidal behaviors, suicidal ideations, suicide attempts, and suspected suicides occurred the least frequently compared to the other drugs. This may suggest that citalopram may have a stronger link to the most severe outcomes, such as completed suicides. At the same time, its lower association with suicidal behaviors, ideations, and attempts could indicate a more selective risk profile, where fewer but more fatal incidents are reported.

### 3.2. Gender and ADRs of the Drugs Studied

A consistent trend observed across all studied drugs was that ADRs were more frequently reported in women. For example, women accounted for 61.3% of the total ADR reports for sertraline and as much as 67% for duloxetine. Our conclusions are consistent with those from another recently conducted study, which also found that ADRs to antidepressants were more frequently observed in women, with a higher incidence rate in females (5.56%) compared to males (3.63%) [45].

This trend underscores the importance of further research into potential gender-specific responses to antidepressants, as women seem more prone to experiencing ADRs. It is possible that biological, psychological, or social factors contribute to this disparity, and addressing this could improve personalized medicine approaches. Biological factors contributing to gender disparities in ADRs include hormonal differences, which can affect drug metabolism, as well as variations in body composition and enzyme activity between males and females. For example, women typically exhibit slower drug clearance rates for certain medications, potentially leading to a higher risk of ADRs. Sociocultural factors also play a role, such as women being more likely to seek medical care and report symptoms compared to men. Studies have shown that women are overrepresented in voluntary pharmacovigilance reports, suggesting a reporting bias in ADR data. These combined factors may explain the observed gender disparities in ADRs

### 3.3. Incidence of Suicide Attempts Across Different Age Groups for Antidepressants

The analysis of suicide attempts in our study across different age groups for six antidepressants reveals that the 18–64 age group consistently exhibited the highest number of cases, indicating that individuals in this demographic are particularly vulnerable to suicide-related ADRs associated with these medications. The drug most frequently associated with suicide attempts among adults up to 64 years of age was venlafaxine (1133 reports). The number of reports describing this adverse reaction was similar for the other drugs, ranging from 522 to 767 reports. Given these findings, it is essential to prioritize targeted monitoring strategies for this high-risk group. Regular follow-ups, especially during the initial weeks of treatment, may help identify early warning signs of adverse reactions. Patient education on potential side effects and the importance of reporting behavioral changes could further mitigate risks. Additionally, alternative treatment approaches, such as psychotherapeutic interventions or the selection of antidepressants with a lower risk profile for suicide-related ADRs, should be considered for individuals with elevated risk factors. Personalized treatment plans that account for the patient’s psychiatric history and risk factors are crucial in minimizing adverse outcomes. Lastly, further research is needed to elucidate the mechanisms that increase susceptibility to suicide-related ADRs in this age group.

In the youngest age group (0–17 years) and the older age group (65–85 years), the frequency of suicide attempts varied by age. In the older age group, suicide attempts were more commonly associated with duloxetine and venlafaxine. Conversely, in the youngest age group, this adverse reaction was more frequently reported with sertraline, followed by fluoxetine, escitalopram, and citalopram. Based on the existing literature, fluoxetine is widely recognized as the safest and most effective antidepressant for children and adolescents, with escitalopram being considered as an alternative when fluoxetine is not tolerated. These drugs have FDA approval for the treatment of depression in children and teens. Given this, special attention should be paid to the choice of antidepressants in younger populations to minimize the risk of psychiatric ADRs, including suicide attempts. Close monitoring during treatment initiation and regular follow-ups are essential for ensuring safety and evaluating the therapeutic response in pediatric patients.

In our study, we observed an intriguing phenomenon where the highest number of suicide attempts in the 65–85 age group was associated with duloxetine and venlafaxine. Considering the more complex mechanism of action of these drugs compared to SSRIs, these findings appear highly logical. Based on this observation, as well as conclusions drawn from the 18–64 age group, it can be hypothesized that the risk of psychiatric ADRs, such as suicide attempts, may be particularly high in these populations when using SNRIs.

Another key observation is the presence of substantial reporting gaps, with a considerable proportion of cases having an unknown age, especially for duloxetine, venlafaxine, and fluoxetine. These gaps complicate the assessment of the actual risk of suicide across different age groups, as the incomplete data prevent a full understanding of the patterns of suicidal behaviors.

### 3.4. Assessment of Suicidal Risks and Reporting Gaps in Antidepressant PhV Data

The analysis of PhV data reveals several important trends in the risk of suicidal behavior, ideation, attempts, and completed suicides across different antidepressants. Venlafaxine stands out as the drug most frequently associated with psychiatric ADRs, completed suicides, and suicide attempts, indicating a higher overall risk profile compared to other antidepressants. Duloxetine, while ranking second in terms of psychiatric ADRs, was the leading drug in causing suicidal behavior and ideation, and it was second only to venlafaxine for suicide attempts. Interestingly, despite this, completed suicides were reported less frequently for duloxetine than for the other drugs, which may suggest that while it may trigger suicidal thoughts and behaviors, these may not always lead to fatal outcomes. It should be noted, however, that the actual number of suicide deaths associated with this drug may be underreported, as in many cases it is unclear what the outcomes of suicidal thoughts and behaviors were (whether they resulted in recovery, hospitalization, or death). This is due to the incomplete nature of many reports. We unfortunately encountered the same issue with the other drugs. While it is clear that in the cases of “completed suicides”, they resulted in death, the final outcome (whether or not the patient died) was not indicated in many reports of “suicidal behaviors”, “suicidal ideations”, and “suicide attempts”. Incomplete reporting hinders the assessment of case severity. Our study highlights the critical importance of ensuring that individuals authorized to report ADRs are aware of the need to specify the outcomes of adverse events, including whether they resulted in death or recovery.

Sertraline is the drug with the highest number of reported ADRs overall (not limited to psychiatric ones), likely due to its status as one of the most frequently prescribed antidepressants and its common use as a first-line treatment for mild to moderate episodes of depression and anxiety disorders. Despite having the highest total number of reported ADRs, psychiatric ADRs were less frequent compared to duloxetine and venlafaxine, and completed suicides accounted for the fewest cases (alongside escitalopram) among all analyzed drugs, representing only 3% of all reported ADRs. However, it should be noted that it was the drug most frequently suspected of causing suicide and was also the second most frequently reported drug to cause suicidal behaviors. The populations treated with sertraline may differ from those treated with duloxetine and venlafaxine, as sertraline is often prescribed for less severe psychiatric conditions. Patients on sertraline may have a lower baseline risk of suicide, influencing the overall patterns of reported ADRs. These findings suggest the need for the careful monitoring of psychiatric ADRs even in medications like sertraline, which are considered safer for psychiatric conditions. Further research is necessary to determine whether the high frequency of suspected suicide reports reflects true drug-related effects or differences in baseline risk and reporting practices.

Citalopram, despite being less frequently mentioned in psychiatric ADRs, was the second most frequent drug (after venlafaxine) to cause completed suicides. This raises the possibility that while citalopram may not trigger a wide range of psychiatric symptoms, it may be more likely to contribute to the most severe outcomes, such as fatal suicides. Its lower association with suicidal behaviors, ideations, attempts, and suspected suicides suggests a more selective risk profile, where incidents are less common but potentially more lethal. This pattern underscores the importance of monitoring for the most severe psychiatric risks, even when a drug has a lower overall frequency of psychiatric ADRs.

Completed suicides were the least frequent with escitalopram, and suicidal ideations as well as suicidal behaviors were also relatively rare. However, escitalopram was still the second antidepressant suspected of causing suspected suicides, highlighting a potential area of concern despite its lower frequency of psychiatric ADRs.

Fluoxetine had the fewest ADRs, both psychiatric and nonpsychiatric, and was never cited as a major contributor to suicide, suicidal behavior, suicidal ideation, or suicide attempts. However, a closer analysis of the percentage of all ADRs represented by completed suicides highlights a more nuanced risk profile. Both citalopram and fluoxetine had the highest relative proportions, with 6% of all reported ADRs for each drug being completed suicides. In comparison, venlafaxine, despite having the highest absolute number of completed suicides, had a lower percentage at 4%. This suggests that when considering the proportional risk relative to overall ADRs, citalopram and fluoxetine might present a more significant concern in terms of completed suicides.

### 3.5. The Impact of Incomplete Outcome Reporting on the Risk Assessment of Suicide-Related ADRs in Antidepressants

One of the most striking findings from our analysis is the high number of reports where the outcomes of suicidal behaviors, suicidal ideations, and suicide attempts remained unknown due to incomplete reporting. For example, duloxetine had the highest number of unresolved cases (3872), which raises concerns about the severity of psychiatric ADRs related to this drug. While venlafaxine had the highest number of completed suicides, it also had 1713 reports where the outcome was unclear, limiting a full understanding of the risk profile.

Interestingly, escitalopram and fluoxetine—despite having fewer total psychiatric ADRs—still presented a significant number of cases with unknown outcomes (1171 and 1097, respectively), which may suggest a potential underestimation of their true risk for severe psychiatric consequences.

This incomplete outcome data make it difficult to accurately assess the real impact of these antidepressants on patients, particularly in terms of fatality or recovery rates. These findings underscore the need for more thorough and detailed reporting in PhV systems to better understand the full extent of psychiatric ADRs and their outcomes across different medications.

Our study demonstrated the implications of incomplete data and highlighted the importance of improving reporting practices for a more accurate risk assessment.

### 3.6. Comparative Risk Assessment of Suicidal ADRs in Antidepressants Using a Six-Level Color-Scale Classification

When comparing the incidence of serious suicidal ADRs using the method we suggested in the form of the six-level color-scale classification, it becomes evident that the most severe ADRs—indicated by brown and red labels—were most commonly associated with the SNRIs (venlafaxine and duloxetine). Among the SSRIs, sertraline stood out, with brown or red labels used twice. However, fluoxetine showed neither brown nor red labels and citalopram and escitalopram had only one red label each, indicating a lower frequency of the most severe suicidal ADRs in these drugs.

Our comparative analysis underscores the varying risk profiles of SNRIs and SSRIs regarding suicidal outcomes, highlighting the need for careful patient monitoring and the consideration of individual drug safety profiles.

### 3.7. Assessing the Link Between Antidepressants and Suicide-Related ADRs: Balancing Data Interpretation with PhV Limitations

As mentioned earlier, one of the major limitations of this analysis is the incompleteness of the reports. In a substantial number of cases, information regarding the final outcome of suicidal behaviors or ideation was not provided. For example, for duloxetine, in 2891 reports of suicidal ideation, the final outcome (whether the patient recovered or died) was not recorded. This lack of data significantly hampers efforts to fully assess the risks associated with these drugs and underscores the need for more thorough and standardized reporting protocols.

Our study showed that certain drugs, such as venlafaxine and duloxetine, are more frequently associated with severe psychiatric ADRs, including suicidal ideation and attempts. However, the interpretation of these findings must take into account the limitations of PhV databases, such as underreporting and the possibility that some psychiatric events may not be directly caused by the drugs but rather by the underlying mental health condition. This is particularly important given that psychiatric ADRs often coincide with the conditions being treated by the medication itself.

The analysis of our study results made us aware of another important limitation when interpreting data from the PhV database. The individual submitting a report on a suspected adverse reaction can list multiple adverse reactions for the same patient, which are then included in one report. For example, if a patient had suicidal thoughts, exhibited suicidal behaviors, and ultimately completed suicide, the reporter would indicate three separate “reactions” in the report, coded in the system as “suicidal ideations”, “suicidal behaviors”, and “completed suicides”. This can distort the overall picture of the actual number of patients who experienced suicidal events. However, it is important to note that all of these reactions are coded under the category of psychiatric disorders, meaning the total number of “psychiatric ADRs” accurately reflects the number of reports for individual patients without duplication.

### 3.8. Implications for Pharmacovigilance Policies

The findings of this study highlight several areas where improvements in PhV policies could enhance drug safety. First, the high rate of missing data in ADR reports underscores the need for stricter requirements and standardized reporting protocols to ensure completeness. Regulatory bodies could mandate the inclusion of key outcome information in all reports, particularly in cases involving psychiatric ADRs. Second, the significant underreporting of ADRs, especially in severe cases like completed suicides, calls for increased awareness and education among healthcare professionals about the importance of PhV reporting. Lastly, the observed discrepancies in ADR reporting across age groups and drug types suggest the need for targeted monitoring strategies, particularly for vulnerable populations such as children, adolescents, and the elderly.

### 3.9. Recommendations for Enhancing Antidepressant Safety and PhV Practices

To improve the safety of antidepressants and address the challenges identified in this study, we propose the following actionable recommendations tailored to clinicians, regulators, and pharmaceutical companies:

For clinicians:

- Implement regular follow-ups, especially during the initial weeks of treatment, to monitor for early signs of psychiatric ADRs, including suicidal ideation or behaviors.
- Provide thorough patient education on the potential adverse effects of antidepressants and emphasize the importance of reporting any behavioral changes or new symptoms.
- Consider patient-specific factors, such as psychiatric history and demographic characteristics, when selecting antidepressants, opting for those with lower risk profiles in high-risk populations.

For regulators:

To enhance the practical utility of PhV data, regulatory authorities should prioritize the following measures:

- Establishing harmonized and standardized reporting protocols across regions to ensure consistency in ADR data collection.
- Introducing mandatory fields in ADR reports, such as patient age, gender, and the final outcome of the adverse reaction, including fatality or recovery to reduce data gaps.
- Developing integrated electronic reporting systems with user-friendly interfaces to encourage reporting by healthcare professionals and patients.
- Promoting public and professional awareness campaigns to highlight the importance of ADR reporting and its role in improving drug safety.
- Encourage national and regional regulatory bodies to develop initiatives aimed at reducing underreporting by increasing awareness and simplifying the reporting process for healthcare professionals.
- For pharmaceutical companies:
- Conduct post-marketing surveillance studies to further evaluate the psychiatric safety profiles of antidepressants, particularly in pediatric and geriatric populations.
- Develop and distribute targeted educational materials to healthcare providers, highlighting the importance of recognizing and reporting psychiatric ADRs.

## 4. Materials and Methods

The analysis was based on data collected in the Eudravigilance system, launched by the EMA to monitor the safety of medicinal products authorized in the EEA. The data used in this analysis include reports submitted to Eudravigilance from its inception in December 2001 to 15 September 2024.

In this study, we analyzed all suspected ADR reports related to antidepressants such as duloxetine, citalopram, escitalopram, fluoxetine, venlafaxine, and sertraline submitted electronically by national regulatory authorities and pharmaceutical companies holding marketing authorizations.

The analysis focused on:

- The total number of reports detailing various ADRs for each of the antidepressant drugs included in the study.
- The frequency of specific ADRs by patient gender for each of the included medications, as well as the number of reports where patient gender information was missing.
- The number of reports describing only psychiatric ADRs for each of the antidepressant drugs analyzed.
- The number of reports describing psychiatric ADRs in the form of “completed suicide”, “suicidal behavior”, “suicidal ideation”, “suicide attempts”, and “suspected suicide” for each of the antidepressant drugs. For the purpose of this analysis, “completed suicides” refer to cases where the report explicitly states that the adverse reaction resulted in the patient’s death due to suicide. In contrast, “suspected suicides” indicate cases where suicide was mentioned as a potential outcome or concern, but the report does not confirm the patient’s death or provide definitive evidence of suicide. This distinction aims to capture both confirmed and potential instances of suicide-related ADRs.
- The proportion of psychiatric ADRs compared to the total number of all adverse reactions (non-psychiatric) for each of the antidepressant drugs studied.
- Evaluating the completeness of reports, specifically whether they contained information on the outcome of the ADRs (whether the reaction resolved, persisted, or led to a serious condition such as hospitalization, disability, or death).
- The source of the reports describing ADRs to the studied drugs, examining whether they were submitted by healthcare professionals (HP), non-healthcare individuals (NHP), or unspecified (NS)—missing information in the report.
- The frequency of suicide attempts associated with the studied antidepressant drugs across the following age groups of 0–17 years, 18–64 years, 65–85 years, and over 85 years, as well as reports where the patient’s age was unknown/not provided.
- The number of ADRs classified as “fatal” (led to the death of the patient) and in how many reports it was unclear whether the ADR resulted in death—marked as “unknown”.

### 4.1. The Importance of PhV Systems for Monitoring ADRs

Pharmacovigilance (PhV) systems are tools used to monitor the safety of drugs after they have been introduced to the market, aiming to detect, assess, and prevent ADRs. In the European Union, this system operates through cooperation between the European Medicines Agency (EMA), the European Commission (EC), and national regulatory authorities responsible for overseeing medicines authorized at the national level. The Eudravigilance system, managed by the EMA, plays a crucial role in collecting ADR data within the European Economic Area (EEA). It allows healthcare professionals and non-healthcare professionals (e.g., patients) to report suspected ADRs to medications. The data gathered by Eudravigilance are analyzed to rapidly identify potential risks associated with drugs, enabling prompt regulatory action, which can include updates to safety warnings or other risk mitigation measures. Safety recommendations developed by the Pharmacovigilance Risk Assessment Committee (PRAC) are a central part of this system. It ensures the continuous monitoring of the safety of medicines on the market, focusing on proactive risk management and patient involvement [46,47].

The analysis of data from EudraVigilance is believed to provide valuable insights into real-world ADRs that may not be apparent in clinical trials due to their limitations. One such limitation is the exclusion of patients who are of very advanced age, who have multiple comorbidities, as well as children and pregnant women. Additionally, there may be factors that are not considered during trials but could increase the risk of adverse drug reactions.

### 4.2. Principles of Operation and Functioning of the Eudravigilance Database

Eudravigilance is a centralized system operated by the European Medicines Agency (EMA) that collects, monitors, and evaluates reports of suspected ADRs within the European Economic Area (EEA). Its primary purpose is to ensure the continuous safety of medicinal products by providing healthcare professionals, pharmaceutical companies, and patients with a platform to report adverse reactions. This system plays a key role in post-marketing surveillance by tracking ADRs that may not have been detected in pre-market clinical trials. When reporting an ADR, certain essential information must be included to ensure the report is comprehensive and can be properly analyzed by the PhV system. The key details that should be provided are [48]:

- Patient Information: This includes the patient’s age, gender, and any relevant medical history or conditions that could influence the reaction. Patient identifiers should be anonymized to protect privacy.
- Suspected Drug: Details about the drug suspected of causing the ADR must be provided. This includes the drug’s name (both generic and brand names), dosage, route of administration, start and stop dates of the treatment, and any batch or lot numbers if available.
- Description of the Adverse Reaction: A clear and detailed description of the ADR should be included. This may encompass the onset date of the reaction, symptoms observed, duration, severity, and any interventions or treatment administered to manage the reaction.
- Outcome: The report should describe the outcome of the adverse event, such as whether the reaction resolved, persisted, or led to a serious condition such as hospitalization, disability, or death.
- Concomitant Medications: It is important to provide information about other medications the patient was taking at the time of the reaction, including OTS drugs, supplements, and any herbal remedies, as these could contribute to or interact with the ADR.
- Reporter Information: The person submitting the report, whether a healthcare professional or a patient, should provide their contact information and their role (e.g., healthcare provider, pharmacist, patient) to allow for follow-up if necessary.

These details help regulatory authorities and healthcare professionals evaluate the potential risks associated with a drug and take necessary actions, such as updating product safety information or issuing warnings. Each adverse reaction reported to Eudravigilance is systematically coded using a standardized medical terminology dictionary, which facilitates easier and more structured searches within the Eudravigilance database for specific side effects related to particular organ systems. For example, within psychiatric adverse reactions, it is possible to search for specific events such as suicide attempts.

### 4.3. Statistical Methods

For the analysis of PhV data related to ADRs associated with antidepressant use, we utilized descriptive statistics to summarize and compare the total number of reported ADRs, focusing on psychiatric ADRs and specific suicide-related events. The following statistical methods were employed:

1. Descriptive Statistics:
  - Frequencies and percentages were calculated to represent the distribution of ADRs by gender, age group, and the source of the report (HP—healthcare professionals vs. NHP—non-healthcare individuals).
  - Proportions were used to illustrate the relative frequency of psychiatric ADRs as a percentage of the total reported ADRs for each antidepressant.

2. Cross-tabulation: Cross-tabulation tables were generated to compare:
  - The frequency of adverse events in the analyzed antidepressant drugs.
  - The frequency of reported suicide attempts across various age groups and between the different antidepressants.
  - The number of reports on suicidal behaviors, suicidal ideations, and suicide attempts where the outcome of the ADRs remains unknown.

3. Trend Analysis: A trend analysis was performed to assess patterns in the reporting of psychiatric ADRs, focusing on the relationship between age, gender, and the occurrence of serious psychiatric outcomes such as suicide attempts and completed suicides.
4. Missing Data Analysis: An analysis was conducted to identify the extent and impact of missing data in the reports, particularly for unknown outcomes in cases of suicidal behaviors, suicidal attempts, and ideations. This step was essential to quantify how incomplete reporting may affect the interpretation of the data.
5. Color-Scale Classification: We developed and applied a six-level color-scale classification in our study to visually differentiate the frequency of ADRs across antidepressants, using colors to represent the relative occurrence of specific adverse events (e.g., suicide attempts, completed suicides), according to a scheme:

Brown—most often reported ADRs among the drugs considered in the study.

Red—very often reported ADRs among the drugs considered in the study.

Orange—moderately reported ADRs among the drugs considered in the study.

Yellow—uncommonly reported ADRs among the drugs considered in the study.

Light yellow—rare ADRs among the drugs considered in the study.

Gray—very rare; the least frequently repeated ADRs among the drugs considered in the study.

6. Worst-Case Scenario Calculation: To estimate the potential true number of ADRs for the selected antidepressants, a worst-case scenario approach was applied. This method assumes that only 6% of all ADRs are reported, based on widely accepted estimates of underreporting in pharmacovigilance systems [34,35,36]. The estimated true number of ADRs (Ntrue) was calculated for each drug using the following formula:
  N_true_ = N_reported_/R
  as follows:

- N_reported_—represents the number of ADR cases reported in the EudraVigilance database.
- R—the reporting rate, assumed to be 6% (0.06) in the worst-case scenario.

By dividing the reported cases by the reporting rate, this method extrapolates the likely scale of ADRs under the assumption of significant underreporting. This approach allows for a systematic estimation of the potential burden of ADRs while acknowledging the limitations inherent in relying solely on voluntary reporting systems.

### 4.4. Limitations of the Dataset

The analysis conducted in this study relies on data collected in the Eudravigilance system, which, while robust, has inherent limitations. These include:

- Underreporting of Adverse Drug Reactions (ADRs): Not all ADRs are reported to the system, which may result in an incomplete dataset. Many ADRs, especially non-severe or transient ones, might not be reported by patients or healthcare providers. Aware of this study limitation, we proposed a worst-case scenario to approximate the true number of psychiatric ADRs associated with antidepressants.
- Reporting Biases: ADR reports often depend on voluntary submissions, leading to potential biases. For instance, careless and chaotic completion of the report.
- Lack of Baseline Patient Data: The reports typically do not include comprehensive information on patients’ baseline mental health status, comorbidities, or prior history of suicidal behavior, which limits the ability to assess causality. Untreated or misdiagnosed comorbidities, such as anxiety disorders, substance use disorders, or chronic pain, could potentially exacerbate the psychiatric adverse effects of antidepressants. For example, the presence of undiagnosed bipolar disorder in patients treated with SSRIs may increase the risk of mania or suicidal ideation. Similarly, untreated chronic pain or the concurrent use of psychoactive substances could amplify the psychiatric burden, complicating the interpretation of ADR data. These factors highlight the complexity of assessing the true impact of antidepressants on psychiatric outcomes and underscore the need for improved reporting systems that include detailed clinical histories.
- Missing or Incomplete Information: A significant number of reports lack crucial details, such as the patient’s age, gender, or the outcome of the adverse reaction. This incompleteness can affect the interpretation and generalizability of findings.
- Non-standardized Reporting Sources: The dataset combines reports from healthcare professionals, non-healthcare individuals, and unspecified sources. This variability in reporting quality can introduce inconsistencies.
- Temporal and Geographic Variability: Reporting practices and regulations may differ across countries and time periods, potentially influencing the dataset’s composition and representativeness.

Despite these limitations, the Eudravigilance database remains a vital tool for identifying real-world safety signals and trends that might not be apparent in controlled clinical trials. Addressing these limitations in future pharmacovigilance studies, such as improving the completeness and standardization of reports, could enhance the utility of such data for public health and clinical practice.

### 4.5. Handling of Missing Data

Missing data in the PhV reports were addressed using descriptive analysis to quantify the extent of missingness. For key variables, such as patient age, gender, and the outcome of the adverse reaction, the proportion of missing data was calculated and reported. Analyses were stratified based on the availability of data to ensure transparency in interpreting the results. A worst-case scenario was applied to provide an approximation of the true number of psychiatric ADRs.

## 5. Conclusions

Our study demonstrated that psychiatric ADRs made up a considerable proportion of the total reported ADRs, ranging from 33.9% to 38.2% for different antidepressants. This underscores the importance of psychiatric monitoring in patients using these drugs, as the frequency of psychiatric ADRs is high. Venlafaxine had the highest number of psychiatric ADRs (13,134 cases), followed by duloxetine (12,790) and sertraline (12,738). However, the analysis revealed that the percentage of psychiatric ADRs relative to total ADRs was highest for duloxetine at 38.2%, followed by fluoxetine at 37.1% and citalopram at 36.4%.

Venlafaxine had the highest number of completed suicides (1635 cases), followed by citalopram (1359), fluoxetine (1296), and duloxetine (1070). However, completed suicides accounted for 3–6% of all reported ADRs, with fluoxetine and citalopram showing the highest percentages (6%).

Our study revealed that ADRs consistently occurred more frequently in women across all studied antidepressants, with the highest proportions observed for duloxetine (67%) and sertraline (61.3%).

The analysis also showed that suicide attempts were most frequent in the 18–64 age group, with notable concerns for the 0–17 age group, especially with sertraline, fluoxetine, and escitalopram, suggesting a need for further research into the safety of these drugs in this age group. However, reporting gaps, particularly regarding unknown patient age, limit the full understanding of suicide-related risks, highlighting the need for more complete data in PhV reporting.

The study highlights the potential and limitations of PhV data in assessing suicide risks associated with antidepressants. The data, although valuable, are significantly affected by underreporting. This suggests that the actual number of psychiatric ADRs, completed suicides, suicidal behaviors, suicidal ideations, suicide attempts, and suspected suicides related to antidepressants could be much higher than what the current data reflect.

Another conclusion that emerged during our data analysis was the incomplete nature of the PhV data, particularly the lack of information on the mental state of patients prior to starting drug therapy. Without knowing whether patients had pre-existing suicidal ideation or behaviors, it is challenging to draw definitive conclusions about whether the antidepressants themselves caused these events.

Our findings also highlight the importance of improving reporting practices, ensuring that outcomes such as recovery or death are consistently recorded to provide a clearer picture of the risks involved in drug use.

Despite the many limitations we encountered while analyzing the PhV data, we believe they provide valuable insights into the real-world risks associated with the use of medications. However, it is essential to strive for these data to be as reliable and accurate as possible by improving the ADR reporting system and implementing solutions that enhance the completeness of ADR reports.

### Key Findings and Practical Implications

The findings of this study provide valuable insights into the patterns, risks, and underreporting of psychiatric adverse drug reactions (ADRs) associated with commonly prescribed antidepressants. By analyzing data from EudraVigilance and applying a worst-case scenario approach, we have highlighted the extent of suicide-related ADRs, disparities across age and gender groups, and the challenges posed by incomplete reporting. Below, we summarize the key observations and propose actionable recommendations to enhance patient safety and pharmacovigilance practices.

Reporting and underreporting of ADRs.

- Only 6–10% of ADRs are reported worldwide.
- Psychiatric ADRs could be significantly underreported. Estimates suggest that the actual number of psychiatric ADRs for the antidepressants analyzed in our study could reach as many as 1,062,550 reports (worst-case scenario analysis).

Psychiatric ADR patterns across drugs.

- Our study demonstrated that psychiatric ADRs constitute 33.9% to 38.2% of all reported ADRs for antidepressants.
- Psychiatric ADRs were more frequently observed with drugs from the SNRI group.
- Compared to the total number of reported ADRs, completed suicides were most frequently observed with citalopram and fluoxetine and least frequently observed with sertraline and escitalopram.

Age and Suicide Attempts.

- Suicide attempts for all drugs were most frequently observed in the 18–64 age group, with venlafaxine leading the cases.
- 0–17 years: Suicide attempts were more common with sertraline, fluoxetine, escitalopram, and citalopram.
- 65–85 years: Duloxetine and venlafaxine were associated with the highest number of suicide attempts.

Gender Disparities:

- For all drugs, ADRs were more frequently observed in women (e.g., 67% of duloxetine ADRs).

Limitations in Reporting:

- The patient’s age was missing in 16–34% of the reports.
- The total number of reports lacking information on whether suicidal behaviors, suicidal ideations, or suicide attempts resulted in death ranged from 808 to 3852.
- The multiplicity of ADRs within single reports complicates interpretation.

Recommendations for:

- Clinicians: Regular follow-ups, personalized treatment, and patient education.
- Regulators: Standardized reporting and initiatives to reduce underreporting.
- Pharmaceutical companies: Post-marketing surveillance and targeted educational efforts.

## Figures and Tables

**Table 1:** Person submitting a report describing the occurrence of ADRs.

Drug	Person Submitting the Report	Total Number Psychiatric ADRs	Completed Suicides	Suicidal Behaviors	Suicidal Ideations	Suicide Attempts	Suspected Suicides
Duloxetine	HP	9141	1006	77	2652	1184	60
	NHP	3639	64	24	1173	175	0
	NS	10	0	0	1	0	0
	TOTAL	12,790	1070	101	3826	1359	60
Venlafaxine	HP	9383	1574	52	944	1407	56
	NHP	3680	51	34	645	167	0
	NS	71	10	1	5	9	0
	TOTAL	13,134	1635	87	1594	1583	56
Citalopram	HP	6113	1274	21	424	841	50
	NHP	1916	68	15	262	76	0
	NS	131	17	0	45	13	0
	TOTAL	8160	1359	36	731	930	50
Escitalopram	HP	5900	767	43	579	1019	67
	NHP	2979	111	23	562	165	0
	NS	28	2	0	7	6	0
	TOTAL	8907	880	66	1148	1190	67
Fluoxetine	HP	6049	1221	54	658	853	63
	NHP	1932	69	29	351	143	0
	NS	43	6	0	2	9	0
	TOTAL	8024	1296	83	1011	1005	63
Sertraline	HP	8284	938	47	794	901	74
	NHP	4312	103	47	557	191	0
	NS	142	8	3	6	31	0
	TOTAL	12,738	1049	97	1357	1123	74

Abbreviations used: NS—not specified; HP—healthcare professionals; NHP—non-healthcare professionals.

**Table 2:** Frequency of ADRs in the analyzed antidepressant drugs.

Summary of Reported Adverse Drug Reactions and Suicidal Events Individual Cases							
Drug	Total Number of Reported ADRs (Individual Cases)	Total Number Psychiatric Adverse Drug Reactions ^A^	Completed Suicides (Fatal) ^A^	Suicidal Behaviors	Suicidal Ideations	Suicide Attempts	Suspected Suicides (Fatal)
Duloxetine	33,438	12,790	1070	101	3826	1359	60
Venlafaxine	36,808	13,134	1635	87	1594	1583	56
Citalopram	22,413	8160	1359	36	731	930	50
Escitalopram	5058	8907	880	66	1148	1190	67
Fluoxetine	21,627	8024	1296	83	1011	1005	63
Sertraline	37,620	12,738	1049	97	1357	1123	74

Explanations used: ^A^ Among them are completed suicides, suicidal behaviors, suicidal ideations, suicidal attempts, and suspected suicides. Frequency of ADRs for the drugs tested: brown—very common; red—common; orange—moderately common; yellow—uncommon; light yellow—rare; gray—very rare.

**Table 3:** Summary of suicide-related reports in the form of suicidal behaviors, suicidal ideations, suicide attempts, suspected suicides, and fatal outcomes associated with antidepressants. Abbreviations and explanations used: NS—not specified. Fatal—the reporter noted in the report that the ADR resulted in the patient’s death.

Drug	Outcome	Suicidal Behaviors	Suicidal Ideations	Suicide Attempts	Total Number of NS ADRs
Duloxetine	Fatal	3	6	11	3852
	NS	60	2891	901	
	TOTAL	101	3826	1359	
Venlafaxine	Fatal	3	31	52	1627
	NS	46	763	818	
	TOTAL	87	1594	1583	
Citalopram	Fatal	0	18	17	808
	NS	13	259	536	
	TOTAL	36	731	930	
Escitalopram	Fatal	4	28	11	1128
	NS	30	423	675	
	TOTAL	66	1148	1190	
Fluoxetine	Fatal	1	12	51	1033
	NS	49	477	507	
	TOTAL	83	1011	1005	
Sertraline	Fatal	3	17	16	1224
	NS	45	583	596	
	TOTAL	97	1357	1123	

**Table 4:** The incidence of suicide attempts in age groups.

Drug	Age					Total
	0–17	18–64	65–85	85+	Unknown	
Duloxetine	29	767	97	6	460 (34%)	1359
Venlafaxine	50	1133	83	5	312 (20%)	1583
Citalopram	74	654	47	6	149 (16%)	930
Escitalopram	175	714	58	4	239 (20%)	1190
Fluoxetine	194	522	39	1	249 (25%)	1005
Sertraline	245	641	55	5	177 (16%)	1123

**Table 5:** Estimated total and psychiatric adverse drug reactions (ADRs) and completed suicides for antidepressants: worst-case scenario analysis.

Drug	Total Number of Reported ADRs (Individual Cases)	Total Number Psychiatric Adverse Drug Reactions ^A^	Completed Suicides (Fatal) ^A^
Duloxetine	557,300	213,167	17,833
Venlafaxine	613,467	218,900	27,250
Citalopram	373,550	136,000	22,650
Escitalopram	417,633	148,450	14,667
Fluoxetine	360,450	133,733	21,600
Sertraline	627,000	212,300	17,483

Explanations used: ^A^ Among them are completed suicides, suicidal behaviors, suicidal ideations, suicidal attempts, and suspected suicides. Frequency of ADRs for the drugs tested: brown—very common; red—common; orange—moderately common; yellow—uncommon; light yellow—rare; gray—very rare.

## Data Availability

The original contributions presented in this study are included in the article. Further inquiries can be directed to the corresponding author.
